# Sex differences in the transition to chronic pain

**DOI:** 10.1172/JCI191931

**Published:** 2025-06-02

**Authors:** Angela F. Smith, Ashley N. Plumb, Giovanni Berardi, Kathleen A. Sluka

**Affiliations:** 1 Department of Physical Therapy and Rehabilitation Science, University of Iowa, Iowa City, Iowa, USA.; 2 Department of Neuroscience, University of Texas at Dallas, Richardson, Texas, USA.

## Abstract

Chronic pain affects more than 50 million Americans, with women disproportionately affected by severe pain, pain interference, and overall disability. The development of chronic pain is multifactorial and often begins with an incident of acute pain associated with an injury or a surgical procedure that transitions to persistent pain lasting for months or years. Despite this, there are limited clinical studies investigating sex differences in predictors and biomarkers for the transition to chronic pain. Several preclinical animal models have been developed to gain a better understanding of the mechanisms for the transition to chronic pain, and several sex-specific mechanisms have been identified across multiple systems. These preclinical models generally involve a multiple-insult approach, in which a priming insult enhances sensitivity to a subsequent induction stimulus. There is emerging evidence from preclinical research for several male-specific and female-specific mechanisms, as well as several studies showing shared mechanisms. Here, we review the clinical and preclinical literature covering sex differences in the periphery and immune system, the central nervous system, and the endocrine system related to the transition to chronic pain. We further highlight gaps in the literature and provide recommendations for future research to understand sex-specific differences in the transition to chronic pain.

## Introduction

Chronic pain affects more than 50 million Americans and is a leading cause of disability, with associated health care and lost-productivity costs totaling $600 billion in the U.S. annually (1). It is associated with reduced productivity and quality of life, which impacts the individual, their family, and society (2). The development of chronic pain is multifactorial; for some individuals there is an absence of clear etiology, while for others it begins with an incident of acute pain associated with an injury or a surgical procedure that transitions to pain and persists for longer than 3 months (3–6). While there are clear sex differences in the prevalence of chronic pain, and accumulating evidence suggests that pain mechanisms across multiple physiological systems are dependent on biological sex, few studies have examined sex differences in predictors or treatments for chronic pain.

A number of prior reviews focused on clinical differences between sexes, suggesting unique sex-specific mechanisms of chronic pain (7–11). However, these have not specifically focused on the acute-to-chronic–pain transition. Therefore, the purpose of this review is to highlight the current understanding of sex differences in the transition to chronic pain in clinical and animal studies, and provide recommendations for future research in the field. We will focus on predictors and risk factors identified in human subjects and underlying mechanisms from animal models of the transition to chronic pain. We use the terms ‘men’ and ‘women’ to describe biological sex for human subjects, which are collected through self report in the available literature. For animal studies, we use the terms ‘male’ and ‘female’ to define biological sex.

## Sex differences in pain

Over 50% of chronic pain conditions are more prevalent in women, whereas approximately 20% of these conditions are more prevalent in men (12, 13). In addition, women are more sensitive to experimental pain stimuli (14), and those with chronic pain often experience more severe pain, pain interference, and widespread pain (15, 16). Overall, women present with greater disability and loss of function due to pain compared with men (15, 17, 18).

It is well known that sex hormones can influence pain in both sexes (19, 20). In both men and women, sex hormone levels peak in the 20–40 year age range. In men, there is a gradual decline in sex hormones with age, whereas women experience an abrupt decrease after menopause. Although the incidence of chronic pain is similar between both sexes prior to puberty (21), the incidence increases in women after puberty and varies through menopause (19, 22). In addition, both clinical and preclinical studies suggest that the prominent sex hormone in males, testosterone, is protective against pain, while mixed effects are noted with the female sex hormone, estrogen (23–27). In conditions such as fibromyalgia, rheumatoid arthritis, and osteoarthritis, lower levels of testosterone are correlated with poorer health status and greater pain severity (28–31). On the other hand, estradiol, the primary form of estrogen in the body during reproductive years, has mixed effects on pain, and these effects may depend on the pain condition (19, 32). Interestingly, perimenopausal women often report greater pain, while postmenopausal women report either increases or decreases in their pain, depending on the condition and intensity of pain (33).

Importantly, there are sex differences in the delivery and effectiveness of treatments for chronic pain (13, 34–42). A secondary analysis of migraine clinical trials using gepants, small molecule calcitonin gene related peptide (CGRP) inhibitors, showed that women experience greater reductions in pain compared with men, and gepants demonstrated effectiveness for acute migraine in women only (43). Sex differences in response to opioids for pain relief have been reported in the literature, suggesting μ-opioid agonists are less potent in women and that women consume less opioids postoperatively; however, these findings are not consistent (7, 34, 44). Metaanalyses show no differences in response to opioids for acute pain, yet this may depend on the opioid used and type of pain treated. Consistently, women used fewer opioids than men for acute pain, and women received lower doses of opioids for chronic pain, particularly those over the age of 45 with the same level of pain (35). It is unclear if these differences for chronic pain are due to a better response to opioids among women or to providers prescribing lower doses to women. For nonpharmacological and interdisciplinary treatments there are reported sex differences, yet these are not consistent among studies, with some showing longer term and greater reduction in men and others showing greater effects in women (37–40, 42). Overall, the majority of clinical studies have not disaggregated or analyzed data by sex, but rather have controlled for sex in the analysis, making it difficult to fully interpret sex differences in response to treatment. Secondary analysis of existing datasets, like that done for gepants, may yield useful data and more definitive results.

## Acute-to-chronic–pain transition incidence and predictors

Across the human lifespan, 20%–70% of individuals will develop chronic pain that persists beyond the usual recovery time from an acute injury, surgical procedure, or illness (45–47). Estimates vary depending on the type of acute injury and the methods used to evaluate pain (48). For example, 6 months after knee replacement surgery for osteoarthritis, 16% of individuals reported pain at rest, while 32% reported pain with movement or activity (49). Similarly, the incidence of chronic postthoracotomy pain at 3 and 6 months ranged from 20%–80%, due to varying methods of pain assessment, as only 2 out of 31 studies used the same approach to measure pain severity (50). Thus, in addition to high-quality clinical trials, use of standardized pain assessments across trials will lead to improved understanding of the acute-to-chronic–pain transition (51).

**Figure 1:**
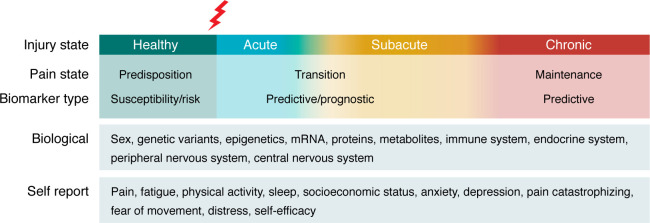
Biopsychosocial factors that could promote the transition to chronic pain in humans. Biopsychosocial factors implicated across the spectrum of acute-to-chronic–pain transition in humans. After an injury during the acute phase and the subacute healing phase, there may be factors that predict who will transition to a chronic pain phase, referred to as predictive and prognostic biomarkers. A number of biological and self-report measures likely contribute to the transition to chronic pain. Sex is routinely a risk factor for the transition to chronic pain and pain severity. However, while a number of studies have investigated factors that promote the transition to chronic pain, few studies have investigated differences between the sexes. There are likely sex differences across biological and psychosocial factors that contribute to the transition to chronic pain, and future studies should examine sex-specific risk and resilience to development of chronic pain.

The underlying mechanisms of the transition to chronic pain are likely multifactorial, involving biological and psychosocial factors, and have been reviewed elsewhere (52) (Figure 1). The transition to chronic pain is strongly predicted by higher levels of pain during the acute phase, the presence of widespread pain, or higher movement-evoked pain (49, 52, 53). However, it is unclear if certain individuals are predisposed to developing chronic pain after an acute injury or if factors associated with acute injury predispose an individual to the development of chronic pain. Understanding the factors that make an individual susceptible or resilient to the development of chronic pain will guide development and implementation of treatments to reduce the risk of acute-to-chronic–pain transition.

There is strong evidence that a number of psychosocial factors predict the transition to chronic pain in a variety of pain conditions, including stress, depression, anxiety, pain catastrophizing, early-life experiences, substance use disorders, and trauma (52, 54, 55). Although the evidence is less substantial than that related to negative psychosocial factors, several studies identified positive psychosocial factors that may reduce the risk for transition to chronic pain, including positive affect, resilience, adaptive coping, engagement with valued activities, and increased physical activity (56–58). Notably, traits associated with positive affect can attenuate biological and/or psychosocial risk factors such as widespread pain, depression, and fear of movement in favor of reduced pain and improved function (59–61).

In contrast to the literature investigating the influence of pain and psychosocial factors on the transition from acute to chronic pain, there has been limited investigation into biological factors or biomarkers. Animal research has driven much of our knowledge; however, there has been an increased focus on conducting mechanistic studies in humans. For example, baseline neuroimaging measures such as white matter fractional anisotropy, gray matter volume of the medial prefrontal cortex, and functional connectivity of the cortico-striatal and hippocampal areas predicted the transition from acute to chronic pain 1 year after an acute back pain episode (62–67). The clinical literature suggests that some genetic, protein, lipid, metabolite, and nervous system factors may relate to the pain experience; however, most of these studies have investigated these markers in isolation and in smaller sample sizes (68–74).

The development of chronic pain is likely multifactorial, resulting from an interaction between biological, psychological, and social factors. Commonly, many risk (priming) factors are present and involve both biological and psychosocial mechanisms. For example, individuals with substance use disorder have increased risk for the transition to chronic pain due to alterations in neurobiological processes, social functioning, and cognitive experiences (54, 75, 76). Similarly, stress induces psychological and physiological changes, perhaps contributing to the uncertainty of cortisol regulation as a biomarker of the development of chronic pain (77, 78) following traumatic injury. Thus, exploration of the numerous biomarkers and factors that contribute to the transition to chronic pain presents significant challenges, and active research in this area is currently underway (52).

## Sex-specific differences in the transition to chronic pain

Despite a large body of literature identifying factors associated with the acute-to-chronic–pain transition, whether sex differences exist among these factors has yet to be elucidated. While over 90% of human studies include both men and women, the majority of clinical pain research does not analyze or disaggregate data by sex (34, 79–86) (Supplemental Table 1; supplemental material available online with this article; https://doi.org/10.1172/JCI191931DS1). Female sex is routinely identified as a predictor for development of chronic pain in adolescents (87–89) but not children (90), suggesting that sex hormones present after puberty play a role in transition to chronic pain (34, 81–86, 91). Further, more women than men developed chronic pain after an emergency department visit for acute pain (15.5% versus 8.7%, respectively) or after thoracic surgery (53.1% versus 38.0%, respectively) (92, 93). Early life experiences involving trauma and stress may have a more pronounced impact on the risk of developing visceral pain with irritable bowel syndrome in women compared with men (94). Thus, women may be more vulnerable to the transition to chronic pain after an acute stressor or injury, which may depend on sex hormones.

Investigations into if psychosocial predictors for the transition to chronic pain differ by sex have been extremely limited. One study found that women have higher levels of pain catastrophizing (characterized by pain magnification, rumination on pain, and pain-related helplessness) compared with men. When reporting pain at a level greater than 1 (on a 0–10 scale), pain catastrophizing predicted development of chronic pain in both men and women; however, when pain was defined as at least 4, pain catastrophizing predicted development of chronic pain only for women (92). Additionally, preoperative chronic pain significantly contributed to postoperative pain in women but not men (92). Whether there are sex differences in other psychosocial predictors remains to be determined.

Thus, emerging evidence suggests that there may be differences in psychosocial predictors of the transition to pain, however, few studies have analyzed clinical data sets for sex, and no studies examined sex differences in biomarkers as a primary aim. Understanding unique predictors of chronic pain between the sexes and the underlying biological and psychosocial mechanisms of chronic pain are critical for tailoring pain management strategies and improving outcomes for both men and women suffering from pain-related disorders across their lifespan. To gain a better understanding of potential sex differences within biological contributors to the transition to chronic pain, we will next discuss the use of animal models that focus on the transition to chronic pain.

## Animal models of the transition to chronic pain

**Figure 2:**
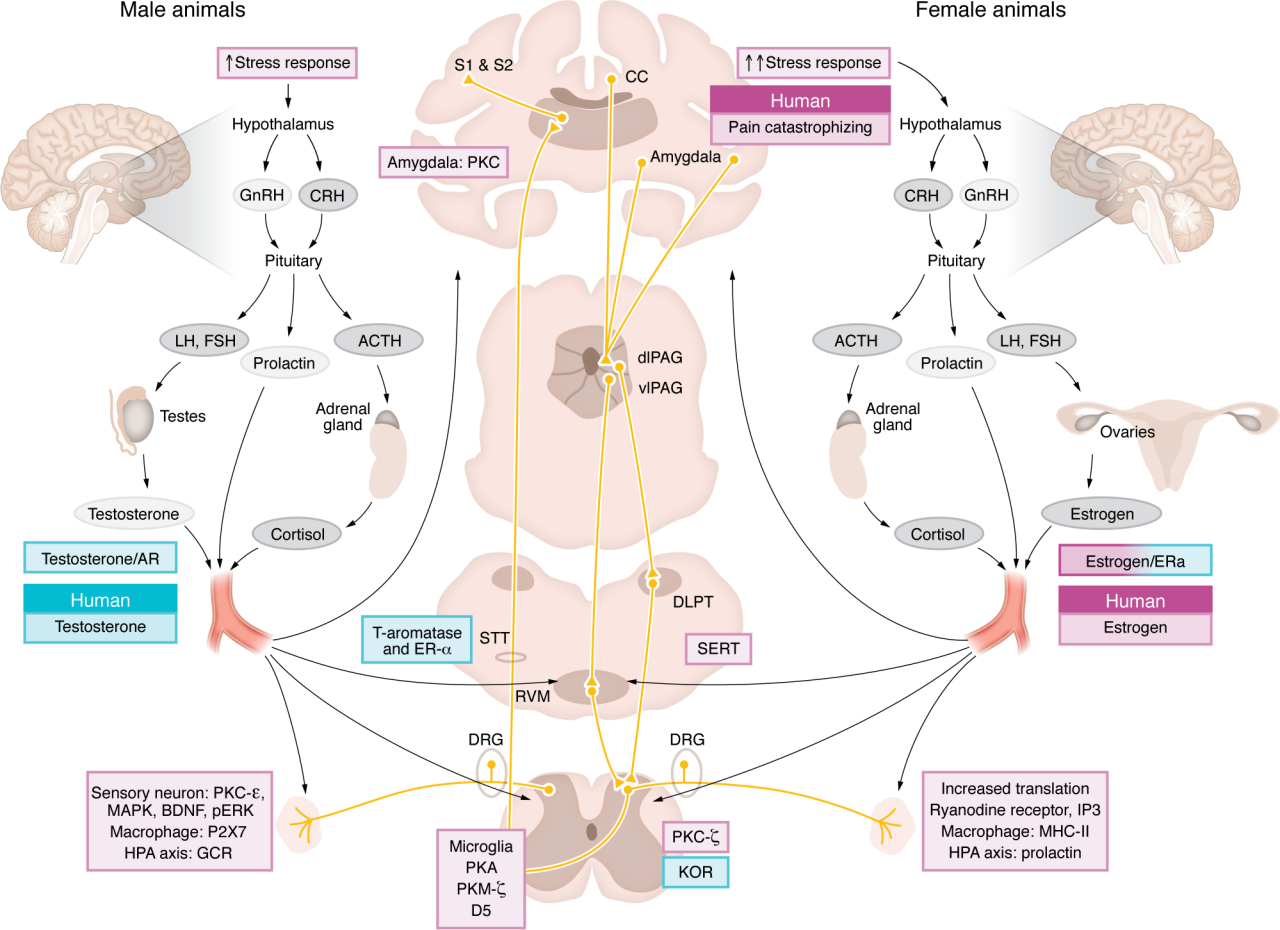
Sex-specific pathways, systems, and mechanisms identified in the transition to chronic pain. Their interaction likely results in a multifactorial process that promotes the transition to chronic pain. Endocrine system release of hormones, including sex hormones, prolactin, and cortisol, into the blood stream can promote this transition via interaction with the nervous and immune systems. In males, testes release testosterone systemically in response to hormones released from the hypothalamus and the anterior pituitary, producing a protective effect through activation of ARs to reduce pain. In females, ovaries release estrogen in response to hypothalamic and anterior pituitary hormonal signals, producing mixed effects that depend on the model. At the site of insult, HPA axis–derived hormones activate intracellular messengers, macrophages, and receptors that can promote the transition to chronic pain, with different mechanisms observed in male versus female animals. Similarly, in the spinal cord, intracellular messengers mediate the transition to chronic pain in a sex-dependent manner, while involvement of microglia and dopamine DRD5 receptors is specific to male mice. In females, tonic KOR activation in the spinal cord protects from development of chronic pain. Unique mechanisms are found supraspinally where, in males, aromatization of testosterone to estradiol activates ER-α, which protects against development of pain, while, in females, increases in SERT promote the transition to chronic pain. PKC in the amygdala plays a unique role in males. While few mechanisms have been identified in humans, roles of testosterone and estrogen generally mirror those found in animals, and pain catastrophizing is more likely to promote the transition in women. Pink boxes indicate risk/transition factors and blue boxes indicate protective factors. Not shown here: mechanisms found in both male and female mice (see Figure 3). GnRH, gonadotropin releasing hormone; CRH, corticotropin releasing hormone; LH, luteinizing hormone; FSH, follicular stimulating hormone; ACTH, adrencorticootropic hormone; GCR, glucocorticoid receptor; STT, spinothalamic tract; DLPT, dorsolateral pontine tegmentum; PAG, periaqueductal gray; CC, cingulate cortex; S1/2, somatosensory cortex 1 and 2.

Animal models of the transition to chronic pain generally involve multiple insults, in which a prior insult or stressor enhances pain sensitivity to a subsequent stimulus, referred to as a priming stimulus and an induction stimulus, respectively. The stimuli are designed to mimic predictors and insults from clinical conditions, including inflammatory stimuli, surgical pain, movement pain, chronic opioid use, early-life insult, or stress (53, 95–103). The priming stimuli create a vulnerability to develop elevated pain behaviors in response to the induction stimuli. The induction stimuli are given after initial hyperalgesia from the priming stimuli resolve and often produce no or little response on their own in unprimed animals but consistently produce a long-lasting, out-of-proportion response in primed animals, thus modeling a chronic pain condition. These models therefore allow for examination of mechanisms underlying the transition to chronic pain, including risk, induction, and resilience factors, as well as exploration of potential novel therapeutics (Supplemental Table 2). Many multiple-insult animal models were initially established and characterized in males and have not been evaluated for behavioral or mechanistic sex differences. However, a few models that demonstrate sex differences in behavior or mechanisms have broadened our understanding of sex differences in the transition to chronic pain (Figure 2).

To mimic acute injury, inflammatory hyperalgesic priming models used carrageenan or CFA as a priming stimulus, which produced hyperalgesia in males, but not females, upon subsequent foot shock (104) or prostaglandin E2 (PGE2) (105). Subsequent studies showed the inflammatory cytokine IL-6 or paw incision as the priming stimulus, followed by PGE2 as the induction stimulus, produced hyperalgesic priming equally in both sexes (106–108). Alternatively, two injections of acidic saline (pH 4.0) into the muscle given 2–5 days apart, but not 10 days apart, produced noninflammatory hyperalgesic priming in both sexes (95, 109–111). As a model of activity-induced hyperalgesia, two intramuscular injections of acidic saline (pH 5.0) five days apart were combined with fatiguing muscle contractions prior to the second injection, none of which produced hyperalgesia alone (96). This model produced sex-specific hyperalgesic priming wherein females developed a more robust, widespread, and longer-lasting hyperalgesia than males. Additionally, females were more susceptible to hyperalgesia in this model, with spatially and temporally remote stimuli capable of inducing hyperalgesia (96). A repetitive ischemia reperfusion injury model used two ischemic insults seven days apart, where the second insult produced longer lasting hyperalgesia than the first. This similarly caused a longer-lasting muscle hyperalgesia in females than males (112). Thus, multiple peripheral insults within a critical window after resolution of initial hyperalgesia from the priming stimulus can produce long-lasting hyperalgesia, some of which present with sex-specific behavioral profiles.

To mimic stress-related priming, repeated stress followed by injection of a nitric oxide (NO donor) in adult mice led to development of migraine-like symptoms in both sexes (102, 113). Repeated stress also induced muscle and visceral hyperalgesia in males, although this was untested in females (114, 115). Early life stress or injury as a priming stimulus can serve as a biologically relevant model of the transition to chronic pain, as infants who experience painful procedures may have increased pain in adulthood (116–120). In animal models, an early life stressor can produce an exaggerated pain response upon reinjury in adulthood (116, 117, 120). Multiple variations of neonatal priming exist, including stress, chemotherapy exposure, inflammation, paw incision, and needle stick, many of which demonstrate a sex difference (103, 121–125). In adolescent mice, chronic alcohol exposure followed by withdrawal produced hypersensitivity in both sexes that is longer lasting than that in adult mice experiencing withdrawal (126). While the extent of priming differs depending on the stimuli used, younger animals are generally more susceptible to priming (53, 116, 117), suggesting that increased plasticity associated with neurodevelopment provides a sensitive window during which mechanisms involved in the transition to chronic pain more readily occur.

Changes in endogenous inhibition in the spinal cord may underly the transition to chronic pain. Latent sensitization models involve a painful insult as a priming stimulus, with blockade of endogenous inhibition, typically opioid receptors, as an induction stimulus after resolution of initial hyperalgesia (127, 128). In this model, spinal or systemic inhibition of μ-opioid receptors (MOR), systemic inhibition of δ-opioid receptors (DOR), or systemic inhibition of μ-δ heteromers reinstated hyperalgesia equally in both sexes. However, spinal inhibition of κ-opioid receptors (KOR) reinstated hyperalgesia more robustly and at lower doses in females than in males (129, 130). Thus, loss of endogenous pain inhibition is one mechanism for the transition to chronic pain with both shared and sex-specific mechanisms.

**Figure 3:**
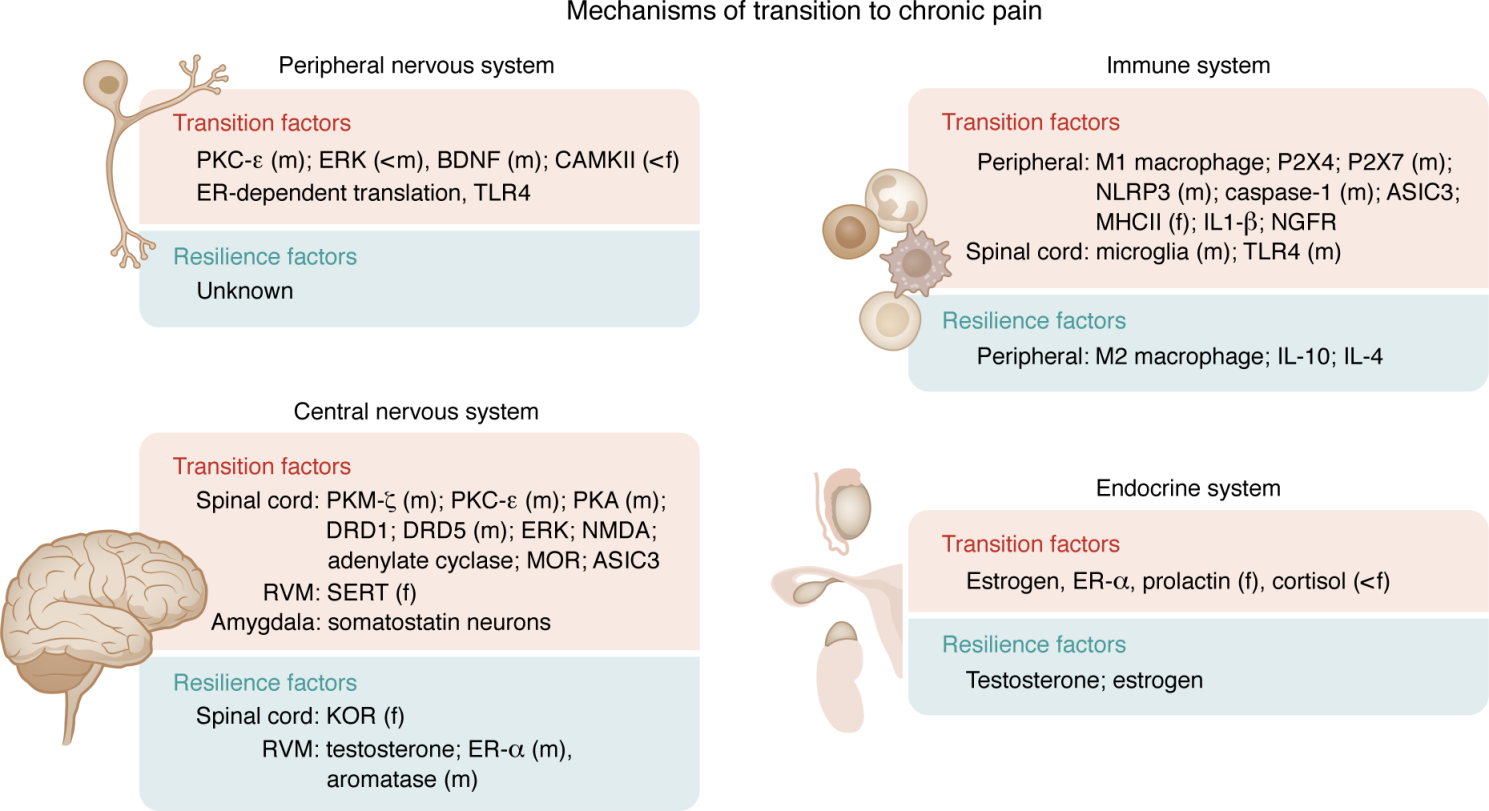
Summary of identified mechanisms involved in promoting and protecting from the transition to chronic pain. Mechanisms identified in animals in the transition to chronic pain from the peripheral and central nervous systems, immune system, and endocrine system. Animal studies have provided evidence of a variety of underlying mechanisms involved in the transition to chronic pain (transition factors) or prevention of chronic pain (resilience factors). Importantly, a number of studies have examined both males and females and identified some sex-specific pathways across all systems. Those with known sex-specific mechanisms are labeled with an “(m)” to show this only occurs in males or an “(f)” to show this mechanism occurs in females. <m and <f indicate that the associated factor contributes to a greater degree in males or females, respectively. It should be noted however, that there are a number of mechanisms that are found in both sexes, and that the sex-specific differences noted may be dependent on the animal model or species used. An overview of the animal literature including mechanism and model details can be seen in Supplemental Table 2. Mechanisms involved in the transition to chronic pain involve peripheral, central, immune and endocrine factors.

A growing body of evidence suggests that multiple priming stimuli can enhance sensitivity to a subsequent induction stimulus to result in long-term hyperalgesia and model the transition to chronic pain, suggesting that the transition to chronic pain is multifactorial. Using these models, sex-specific mechanisms have been shown in the periphery and immune system, the central nervous system, and the endocrine system (Figure 2); however, it should be pointed out that there are also shared mechanisms between the sexes (Figure 3). Understanding mechanisms that contribute to the transition to chronic pain in both sexes is critical to development of strategies to prevent chronic pain conditions. Thus, this review focuses on studies that examine mechanisms during the priming and induction of chronic pain, rather than those that reverse the hyperalgesia once developed.

## Peripheral neural and immune mechanisms in animal models

It has become increasingly clear that peripheral neural and immune mechanisms are involved in the transition to chronic pain (131, 132). Intracellular messengers can modify receptor function and initiate gene transcription and protein translation to produce long-lasting changes in neuronal excitability. At the level of the nociceptor, there is substantial evidence for the intracellular messenger PKC-ε in the generation of hyperalgesic priming. Priming with a PKC-ε activator produced an exaggerated pain response to PGE2 injection in males but not females (105). However, priming with activators of intracellular messengers downstream of PKC-ε — IP3, CAMKII, and ryanodine receptors — produced hyperalgesic priming to PGE2 in both sexes, although at lower doses in females (133–136). Inhibition of the intracellular messengers — PKC-ε, MEK, ERK, and CAMKII — during induction prevented hyperalgesic priming in both sexes (136). In a hyperalgesic priming model that uses chronic opioid agonism as the priming stimulus, coinhibition of MAPK and Src reversed priming in males but not females (99). In parallel, there was increased activation of ERK (measured as pERK) in sensory neurons in a noninflammatory hyperalgesic priming model to a greater extent in males than in females (137). AU-rich element RNA-binding protein, which regulates translation, was more highly phosphorylated in females than males in a repeated ischemia reperfusion injury model; inhibition in females attenuated hyperalgesia, while overexpression in males potentiated hyperalgesia (112). In contrast, local (paw) inhibition of translation reduced ryanodine hyperalgesic priming in both sexes (136). Thus, in the periphery, priming may produce changes in intracellular messengers that modulate production of proteins to make nociceptors more sensitive to a subsequent stimulus and result in hyperalgesia, with some sex-specific mechanisms.

Brain-derived neurotrophic factor (BDNF) plays a significant role in hyperalgesic priming in a sex-specific manner. Peripheral blockade of BDNF during induction in the IL-6 or activity-induced hyperalgesic priming model prevented hyperalgesia in males but not females (138, 139). However, in activity-induced hyperalgesic priming, BDNF was upregulated in the DRG of both males and females, suggesting that BDNF may play a role in females, even if blockade is not sufficient to prevent hyperalgesia (138). Surprisingly, in IL-6 hyperalgesic priming, this sex difference for BDNF was only present in mice; blockade of BDNF prevented IL-6 hyperalgesic priming in both sexes in rats (139). Together these data support that BDNF contributes to hyperalgesic priming in males, and may play some role in females, although the data is mixed.

In activity-induced hyperalgesic priming, males and females present with both shared and unique immune mechanisms in the development of hyperalgesia. In both sexes, depletion of macrophages in muscle prevented the development of hyperalgesia, showing a role for local macrophages in induction of priming (96, 140, 141). However, blockade of the immune purinergic receptor P2X7 and its downstream pathway (NLRP3/caspase-1) in muscle during induction reduced hyperalgesia in males but not females (140). Despite this behavioral sex difference, there was an equivalent upregulation of genes in the P2X7 pathway in both sexes, suggesting alternative mechanisms or a suppression pathway in females (140). On the other hand, in females, but not in males, there was an upregulation of MHC II, a cell-surface signaling molecule expressed in macrophages, and blockade of MHC II during induction prevented activity-induced pain (142). Not all pathways present sex differences, as blockade of IL-1β, P2X4, and ASIC3 in muscle prevents activity-induced hyperalgesic priming in both sexes (140, 141, 143). Similarly, in a model of neonatal incision priming, macrophage-deficient mice of both sexes did not develop hyperalgesia to a second incision in adulthood (123). Further, adult mice that received neonatal incision had altered macrophage epigenetic and mRNA signatures, including for nerve growth factor receptor (NGFR), and macrophage-specific NGFR knockout attenuated the hyperalgesic priming to a second incision given in adult mice (123). These studies highlight how alterations in the immune system, particularly macrophages, can influence the transition to chronic pain with both sex-specific and shared mechanisms.

While scientists generally examine peripheral mechanisms that promote a transition to chronic pain, it should be noted that there may also be factors that promote recovery. Indeed, we have shown that regular exercise prevents the transition to chronic pain in both male and female mice through multiple mechanisms (144–148). While sex-specific factors clearly can modulate the transition to chronic pain, there are likely sex-specific resilience factors yet to be discovered.

## Central neural and immune mechanisms in animal models

Sites within the central nervous system are involved in nociception and pain, some of which have been investigated for sex differences in the transition to chronic pain (149) (Figure 2). The spinal cord has been extensively studied as it receives nociceptive input from injured sites and integrates pain modulation from supraspinal sites. Additionally, the amygdala has emerged as a key area in the emotional aspects of pain, and recent work shows its involvement in the transition to chronic pain. Lastly, the thalamus, which relays nociceptive information from the spinal cord to the cortex, has also been investigated for its role in the transition to chronic pain. Within each of these sites, identified sex-specific mechanisms in the transition to chronic pain are discussed below.

### Spinal cord.

Intracellular signaling in the spinal cord plays a role in the transition to chronic pain by enhancing long-term changes in neuronal excitability through modification of receptors and increases in gene transcription. In noninflammatory hyperalgesic priming, knockout or inhibition of protein kinase M-ζ (PKM-ζ) in the spinal cord prevented chronic pain in male but not female mice (150). Targeting protein kinase A (PKA) in the same noninflammatory priming model also prevents development of pain in males but not females (151, 152). Similarly, in the latent sensitization model, reinstatement of hyperalgesia after blockade of KOR was prevented by spinal inhibition of PKA, and PKA activation alone reinstated hyperalgesia in male but not female mice (153). As in the periphery, in female mice, blockade of protein translation prevented IL-6 hyperalgesic priming in males but not females (106). Taken together, these studies suggest that, within the spinal cord, intracellular mechanisms are differentially involved in pain in males and females at different time points in the transition to chronic pain.

Spinal dopamine both inhibits and facilitates pain (154). In IL-6 hyperalgesic priming, spinal blockade of both dopamine D1 and D5 receptors delayed the transition to chronic pain in both sexes, while a D5 receptor knockout prevented development of hyperalgesic priming only in males (107, 155). This suggests that dopamine D1/D5 receptors differentially contribute to the development of chronic pain in males and females. In contrast, in neonatal incision priming, dopamine D1/D5 receptors contributed to increased long-term potentiation (a measure of central activity) in the spinal cord (156, 157). In D5-knockout mice, facilitation of long-term potentiation was equally reduced in both sexes (157). These data suggest that spinal dopamine plays a role in the transition to chronic pain by reducing central excitability in both sexes, and D1 and D5 receptors may have a sexually dimorphic role.

Within the spinal cord, microglia activation in male mice plays a role in hyperalgesia in a variety of animal models of pain (158–161). In animals with neonatal incision priming, inhibiting microglia at the time of neonatal incision prevented hyperalgesic priming to adult reincision and reduced expression of genes associated with microglia proliferation in the spinal cord in adult males but not females (103, 124). Similarly, spinal blockade of the microglial purinergic receptor P2X4 prevented IL-6 hyperalgesic priming in only males (162). Thus, spinal microglia appear to play a more consequential role in the transition to chronic pain in males.

### Supraspinal.

Compared with research in the spinal cord, less work has investigated potential supraspinal mechanisms underlying the transition to chronic pain, particularly for sex differences. Like in the peripheral nervous system and spinal cord, intracellular messengers in the amygdala have been implicated in the transition to chronic pain. In an incision hyperalgesic priming model, there was increased expression of PKC-ζ in the basolateral amygdala, while inhibiting atypical PKC or genetic deletion of PKC-ζ prevented hyperalgesia to PGE2 after plantar incision in male, but not in female, mice (108). In the latent sensitization model, systemic naltrexone increased Fos expression in the central nucleus of the amygdala similarly in both sexes, a portion of which express PKC-δ, showing increased activation of the amygdala in primed animals (163). Thus, PKC isoforms within the amygdala appear to play a role in the transition to chronic pain, similar to that observed in sensory neurons and the spinal cord, some of which are sex dependent.

As noted for the periphery and spinal cord, not all pathways show sexual dimorphism. In the amygdala, inhibition of GluA2 prevented hyperalgesic priming following paw incision in both sexes (108), chemogenic inactivation of somatostatin-expressing neurons during priming prevented development of noninflammatory hyperalgesic priming (164), and microinjection of a competitive MOR agonist into the CeA reinstated hyperalgesia in both sexes (163). In the paraventricular thalamus, Ca_v_3.2-dependent activation of ERK during the priming phase, but not during the maintenance phase, was necessary for initiation of the noninflammatory hyperalgesic priming model in both sexes (165). Thus, emerging reports suggest that the amygdala and the paraventricular thalamus are involved in the transition to chronic pain, with some pathways and receptors showing sexual dimorphism that varies depending on brain region, pathway, and model.

## Endocrine mechanisms in animals

The endocrine system is increasingly being recognized for its role in pain. The endocrine system is made up of multiple glands that release hormones that can produce effects throughout the body (166, 167) (Figure 2). Researchers studying preclinical sex differences in pain have primarily focused on sex steroids produced by the gonads, in particular, circulating testosterone in males and estrogen in females and the hypothalamic-pituitary-adrenal (HPA) axis.

## Sex hormones

### Testosterone.

The male sex hormone, testosterone, has routinely been shown be protective in pain models, including hyperalgesic priming models. In males, removal of endogenous testosterone with a gonadectomy prolonged hyperalgesic priming in some models (IL-6 and activity-induced hyperalgesic priming) (24, 106), but had no effect in the inflammatory hyperalgesic priming model (105). On the other hand, testosterone administration to females attenuates hyperalgesic priming in the activity-induced pain model (24). In both males and females, exercise protects against development of activity-induced hyperalgesic priming through androgen receptor–mediated mechanisms (145). This suggests that testosterone protects against the development of pain in both sexes, and sex differences in the transition to chronic pain may be due, in part, to different levels of circulating testosterone between the sexes.

One region shown to be involved in this protection is the rostral ventromedial medulla (RVM). In the RVM of males, testosterone is aromatized into estradiol to activate estrogen receptor α (ER-α) and protect against the transition to chronic pain. However, blockade of ER-α in the RVM in females had no effect on activity-induced hyperalgesic priming (168). In females, exogenous testosterone reduced the increases in the serotonin reuptake transporter (SERT) normally observed in the RVM in the activity-induced priming model (169). Thus, unique pathways within the RVM mediate the protective effects of testosterone on hyperalgesic priming in males and females.

### Estrogen.

The female sex hormone estradiol has been extensively studied in hyperalgesic priming and shows mixed results. In animals with carrageenan or PKC-ε hyperalgesic priming, administration of estradiol suppressed hyperalgesia in males, while ovariectomy or ER-α inhibition facilitated hyperalgesic priming to PGE2 in females (105, 135). In contrast, ovariectomy attenuated IL-6 or noninflammatory hyperalgesic priming (106, 110) but had no effect in the activity-induced priming model (96). These data suggest that estrogen may be protective in males, yet its effects are highly model dependent in females.

A few studies have examined if the mechanisms underlying the transition to chronic pain depend on estrogen or activation of estrogen receptors. Females were more sensitive to priming by the intracellular messengers ryanodine or IP_3_, an effect regulated by ER-α (133). Further, local blockade of translation prevented hyperalgesic priming in females that was abolished by ovariectomy, but had no effect in males (106). Thus, estrogen may modulate some of the female-specific mechanisms observed in hyperaglesic priming models.

## HPA axis

The HPA axis is a key part of the body’s stress response, releases cortisol, and has been implicated in chronic pain (170). Interestingly, in animals primed with the chemotherapy drug oxaliplatin, PGE2-induced hyperalgesia was reduced by adrenalectomy; however, only male rats were tested (171). In this hyperalgesic priming model, reduction of β2-adrenergic receptors or glucocorticoid receptors prevented the hyperalgesia, suggesting a role for the HPA axis (171). Similarly, in a repeated stress migraine model (combining repeated stress with NO donor injection), blockade of corticosteroid production or glucocorticoid receptors prevented hyperalgesia in both sexes, while repeated corticosterone injections reproduced migraine behaviors only in female mice (113). These data show a role for the HPA axis in the transition to chronic pain, yet whether there are sex-specific mechanisms are yet to be determined.

Prolactin, a neuroendocrine hormone secreted from the pituitary that can modulate the HPA axis, is involved in hyperalgesic priming (106). Specifically in females, but not in males, priming with prolactin produced prolonged hypersensitivity to PGE2, while prolactin receptor knockout in sensory neurons prevented IL-6 hyperalgesic priming. Interestingly, chemical removal of the pituitary had no effect on hyperalgesia following induction with PGE2 in females and slightly prolonged the response in males (106). These data suggest that local synthesis of prolactin and its receptor play a critical role in sensory neurons in the transition to chronic pain.

As is the case in the peripheral nervous and immune systems, efforts often focus on mechanisms that contribute to the transition to chronic pain. However, neonatal handling produces resilience to oxaliplantin and stress priming in males, which is dependent on testosterone and adrenal gland signalling (171, 172). Thus, the endocrine system, particularly testosterone and activation of the HPA axis, play a critical role in the resilience to development of chronic pain, and future studies in this area may identify shared and sex-specific resilience mechanisms.

## Outlook on sex-dependent factors in chronic pain

As highlighted in this review, there is limited understanding of the mechanisms and factors leading to the transition from acute to chronic pain, particularly in humans. Emerging evidence shows that some predictors and mechanisms may be sex specific and that sex-specific mechanisms may depend on the pain condition (Figure 3). Indeed, the periphery and immune system, the central nervous system, and the endocrine system all play a role in the sexual dimorphism in the transition to chronic pain and likely act together to promote the transition to chronic pain (Figure 2). Understanding these sex-specific mechanisms could lead to improved pain management using a more individualized approach. Below, we outline a number of potential pathways to improve our understanding of sex-specific mechanisms not only in the transition to chronic pain, but for the scientific community as a whole.

First, sex as a biological variable needs to be routinely considered in both preclinical and clinical studies investigating the transition to chronic pain; specifically, sex needs to be factored into research design, analysis, and reporting in cell culture, animal, and human studies. While funding agencies across the globe (e.g., the U.S., Europe, Canada, and Australia) mandated nearly a decade ago that all research grants consider sex as a biological variable, the most recent literature reviews in the journal *PAIN* show that only about 50% of animal studies include males and females, and less than 20% of clinical studies report results by sex (169). Beyond the requirements of funding agencies, it will take a cultural change in scientific research to fully implement this approach. Reviewers of grants and manuscripts need to be aware of the increased workload that is required for basic science and clinical studies that are powered to examine sex-specific differences and scale their expectations appropriately. Funding agencies need to enforce the inclusion of and support the reporting of sex-specific differences. Importantly, we believe that the biggest impact to promote change will come from scientific journals. Journals should require the use of sex as a biological factor in both basic science and clinical studies, and, unless adequately justified, should encourage the reporting of effects by sex or sharing of all sex-specific data.

Second, understanding sex as a biological variable in both behavioral output and mechanisms is critical to fully understand the transition to chronic pain and to ultimately provide individualized patient-centered care. Given the variability of underlying biological and psychosocial mechanisms, it will be increasingly important to power studies to detect sex-specific effects. This means that the scientific community will need to recalibrate its expectations about what should be included in an experimental design. For example, we need to understand how sex differences affect changes in pain across the lifespan and uncover sex-specific biopsychosocial mechanisms to develop tailored treatments for males and females with chronic pain. These differences may affect observed sex biases across pain management and science (79, 173, 174).

Last, in chronic pain research, the use of large datasets, like the Acute to Chronic Pain Signatures (A2CPS), the UK Biobank, or the National Health and Nutrition Examination Survey (NHANES), are ideal for performing high-powered, sex-specific analyses; any study using these data should routinely perform an analysis by biological sex. As an example, the A2CPS is a unique dataset that focuses on the transition from acute to chronic pain in men and women following surgery (175). The A2CPS research initiative collects biomarkers before and after surgery and examines them for factors that predict the transition to chronic pain 6 months later. Biomarkers and predictors are collected across multiple domains, including pain and associated symptoms; psychosocial measures; brain imaging; quantitative sensory testing; and a variety of omics platforms (e.g., genetic variants, extracellular RNA, proteins, lipids, and metabolites). Importantly, this dataset will be available to the public for future research analyses. Using a large dataset that includes data for both men and women will allow future studies to examine for sex-specific differences in mechanisms of chronic pain to generate novel hypotheses.

## Supplementary Material

Supplemental data

## Version 1 06/02/2025

Electronic publication
