# Supplementary material for: Sex differences in the transition to chronic pain
Source: J Clin Invest. 2025 Jun 2;135(11):e191931. doi: 10.1172/JCI191931 (PMC12126234; doi:10.1172/JCI191931)
Supplement: Supplemental data [file jci-135-191931-s304.pdf]

Citations correspond to the reference list in the main text.

Supplemental Table 1. Studies investigating human sex differences in acute-to-chronic pain transition.

Aim / Scope	Sex Difference	Reference
Neck and Low Back Pain		
Transition to chronic low back pain	Data not Disaggregated by sex. Sex was entered as a variable in the multivariate analysis however, not a significant factor in predicting the development of chronic low back pain regardless of risk stratification (low-, medium-, or high-risk	(82)
Predictors of moderate or severe neck pain following whiplash injury	Data not Disaggregated by sex. Female sex increased risk of moderate to severe neck pain following whiplash among nonlitigants, but not among litigants.	(83)
Psychosocial - Pain Catastrophizing		
Differences in the relation of pain catastrophizing to chronic pain between sexes in patients presenting to the emergency department	Females had greater depression, chronic pain in other locations, and pain catastrophizing. Pain catastrophizing increased the risk of chronic pain in both males and females when chronic pain was defined as $\geqq 1$ on a 0-10 numerical rating scale at 90 days. When chronic pain was defined as $\geqq 4$ at 90 days, pain catastrophizing was associated with chronic pain in females.	(93)
Pain Sensitivity		
Acute to chronic pain sensitization using thermal and ischemic stimuli	Pain ratings and stress ratings disaggregated by sex. Conditioned pain hypersensitivity present in males but absent in females.	(178)
Factors associated with medical/pharmacological analgesia		
Factors associated with patient-controlled analgesia.	Data not Disaggregated by sex. Sex was not a significant factor except in patients with lower abdominal surgery.	(84)

Differences in opioid-induced analgesia or antinociception among sex	Data not Disaggregated by sex. Women display greater opioid analgesia than men.	(35)
Prediction of Postoperative Pain		
Prediction of postoperative pain	Data not Disaggregated by sex. Female sex was associated with a better global recovery.	(85)
Prediction of postoperative pain	Data not Disaggregated by Sex. Females at greater risk of developing early severe post-operative pain.	(86)
Prediction of postoperative pain	Data not Disaggregated by Sex. Post-operative pain was positively associated with female gender	(92)
Prediction of postoperative pain	Most Data not Disaggregated by Sex. % of males and females reporting severe pain vs without severe pain is presented. Significantly more male patients suffered from severe pain than female patients. Many female patients undergoing gynecological procedures had a lower incidence of pain than did male patients undergoing orthopedic procedures.	(87)
Prediction of chronic postsurgical pain	The incidence of chronic postsurgical pain is higher in women after thoracotomy. Women's mental state, depression, and catastrophizing scores were worse at baseline. Presurgical pain prevalence was higher in women. Pain intensity after thoracotomy did not differ between men and women. Mental scores worsened in men with chronic pain but improved in women.	(94)

Supplemental Table 2: Studies investigating sex differences in animals

Mechanism	Region	Male Findings	Female Findings	Model(s)	Species	Reference(s)
Acid Sensing Ion channel 3	Systemic	Global KO but not cKO on primary afferents prevented development of hyperalgesia	Global KO but not cKO on primary afferents prevented development of hyperalgesia	Hyperalgesic priming (pH 5.0, fatiguing muscle contractions + pH 5.0)	Mice	(144)
Physical Activity	Systemic	Voluntary running prevented hyperalgesia, reduced SERT expression in RVM, promoted anti-inflammatory macrophage phenotype. Resistance training prevented priming and reversed hyperalgesia	Voluntary running prevented hyperalgesia, reduced SERT expression in RVM, promoted anti-inflammatory macrophage phenotype. Resistance training prevented priming but did not reverse hyperalgesia when started after model induction	Hyperalgesic priming (pH 4.0, pH 4.0) or (pH 5.0, fatiguing muscle contractions + pH 5.0)	Mice	(143,146-149)
Testosterone	Systemic	Reduced pain behaviors, orchiectomy facilited pain, exercise protected again pain through androgen receptors	Exogenous testosterone reduced pain, exercise protected against pain through androgen receptors	Hyperalgesic priming (pH 5.0, fatigueing muscle contractions + pH 5.0) or (IL-6, PGE2)	Mice	(25,107,147)
Calcitonin Gene Related Peptide	Systemic	Antibody blocked migraine like phenotype	Antibody blocked migraine like phenotype	Migraine hyperalgesic priming (repeated stress + NO donor)	Mice	(103)
Cortisol	Systemic	Reduction or receptor blockade prevented priming. Injection did not induce hyperalgesia.	Reduction or receptor blockade prevented priming. Injection induced hyperalgesia.	Migraine hyperalgesic priming (repeated stress + NO donor)	Mice	(114)

Protein Kinase C Epsilon	Peripheral	Agonist induced priming, estrogen implantation prevented priming	Agonist did not induce priming, ovariectomy allowed priming to occur	Hyperalgesic priming (carrageenan, PGE2)	Rats	(98,106)
Ryanadine Receptors	Peripheral	Agonist induced priming	Agonist induced priming at a lower dose than in males, blocked by ERa antagonist	Hyperalgesic priming (ryanadine, PGE2)	Rats	(134,136)
Peripheral Inflammation	Peripheral	CFA or carrageenan induced priming. Neonatal needle stick induced priming to adulthood CFA	CFA or carrageenan did not induce priming. Neonatal needle stick did not induce priming to adult CFA	Hyperalgesic priming (CFA, chemical challenge/footshock) or (carrageenan, PGE2) or (neonatal heel stick, CFA)	Mice, rats	(105,106,126)
Muscle Fatigue	Peripheral	Fatigue and muscle insult led to local, short term hyperalgesia following priming	Fatiuge and muscle insult led to bilateral, long term hyperalgesia following priming, no effect for OVX	Hyperalgesic priming (pH 5.0, fatiguing muscle contractions + pH 5.0)	Mice	(97)
Nerve Growth Factor Receptors	Peripheral	Increased mRNA after priming, knockout on macrophages prevented hyperalgesia	Increased mRNA after priming, knockout on macrophages prevented hyperalgesia	Hyperalgesic priming (neonatal incision, adult reincision)	Mice	(124)
Prolactin	Peripheral Sensory Neurons	Knockout or blockade did not prevent priming	Knockout or blockade prevented priming. Systemic blockade prevented hyperalgesia.	Hyperalgesic Priming (IL-6, PGE2) or Repeated ischemic reprofusion	Mice	(107)

mRNA Translation	Peripheral Sensory Neurons	Overexpression of AU-rich element binding protein induced hyperalgesia, lower expression after pain than in females. Local inhibition of translation prevented hyperalgesia.	Knockdown of AU-rich element binding protein induced hyperalgesia, higher expression after pain than in males. Local inhibition of translation prevented hyperalgesia.	Repeated ischemic reprofusion or Hyperalgesic Priming (Ryanodine, PGE2)	Mice	(113,137)
Bone Derived Neurotrophic Factor	Peripheral Sensory Neurons	Blockade in spinal cord or muscle delayed or prevented hyperalgesic priming. Upregulated in DRG neurons after pain	Blockade in spinal cord or muscle did not delay or prevent hyperalgesic priming. Upregulated in DRG neurons after pain	Hyperalgesic priming (pH 5.0, fatiguing muscle contractions + pH 5.0) or (IL-6, PGE2)	Mice	(139,140)
Calcium/calmodulin-Dependent Protein Kinase II	Peripheral Sensory Neurons	Activation induced priming and transition to chronic pain, blockade prevented priming and transition to chronic pain	Activation induced priming and transition to chronic pain, blockade prevented priming and transition to chronic pain	Hyperalgesic priming (CamKII or Ryanadine Receptor, PGE2)	Rats	(135,137)
Extracellular Signal Regulated Kinase	Peripheral Sensory Neurons	Bilateral increase in pERK after pain, greater than in females	Unilateral increase in pERK after pain, less than in male.	Hyperalgesic Priming (pH 4.0, pH 4.0)	Rats	(138)
Opioid Receptors	Peripheral Sensory Neurons	MOR knockout potentiated hyperalgesia, systemic MOR inhbition in KOs did not reinstate pain.	MOR knockout potentiated hyperalgesia, systemic MOR inhbition in KOs weakly reinstated pain.	Latent sensitization (hindpaw inflamation, opioid block)	Mice	(99)
P2X7	Muscle	Blockade of P2X7 and downstream signalling molecules delays hyperalgesia, these targets are upregulated in pain	Blockade of P2X7 and downstream signalling molecules does not delay hyperalgesia, but these targets are upregulated in pain	Hyperalgesic priming (pH 5.0, fatiguing muscle contractions + pH 5.0)	Mice	(141)
P2X4	Muscle	Upregulated following pain; blockade or knockdown prevents	Upregulated following pain; blockade or knockdown prevents development of hyperalgesia	Hyperalgesic priming (pH 5.0, fatigueing muscle	Mice	(145)

		development of hyperalgesia		contractions + pH 5.0)		
Major Histocompatibility Complex Class II	Muscle	mRNA not upregulated after pain, antibody does not impact pain behavior	mRNA upregulated after pain, antibody reduce pain behavior	Hyperalgesic priming (pH 5.0, fatiguing muscle contractions + pH 5.0)	Mice	(143)
Toll Like Receptor 4	Muscle	Inhibition prevents priming but not induction	Inhibition prevents priming but not induction	Hyperalgesic Priming (pH 4.0, pH 4.0)	Mice	(96)
Spike timing dependent long term potentiation	Spinal cord	Neonatal incision increases suseptability to spike timing dependent long term potentiation by relaxing timing rules and recruiting AMPA receptors	Neonatal incisionn increases suseptability to spike timing dependent long term potentiation by relaxing timing rules and recruiting AMPA receptors	Hyperalgesic priming (neonatal incision, neonatal incision)	Mice	(158)
Protein Kinase A	Spinal Cord	Blockade delays/prevents hyperalgesia	Blockade does not prevent priming or reinstatement of pain	Hyperalgesic Priming (pH 4.0, pH 4.0), Latent sensitization	Mice, rats	(153-155)
Protein Kinase C	Spinal Cord	Blockade does not delay hyperalgesia	Blockade prevents priming	Hyperalgesic Priming (pH 4.0, pH 4.0)	Mice, rats	(154,179)
Microglia	Spinal Cord	Pharmacological inhibition during neonatal incision reduces hyperalgesia in adulthood, inhibition during priming prevents induction.	Pharmacological inhibition during neonatal incision does not reduce hyperalgesia in adulthood. inhibition during priming does not prevent induction.	Hyperalgesic Priming (neonatal incision, adult reinincision or IL-6, PGE2)	Mice, rats	(104,125,164)
Dopamine Receptors	Spinal Cord	DRD5 knockout prevents hyperalgesia at second insult, mimics intrathecal D1/D5 blockade, D1/D5 facilitate long term potentiation	DRD5 knockout does not impact hyperalgesia at the second insult, but intrathecal D1/D5 blockade delays hyperalgesia, D1/D5 facilitate long term potentiation	Hyperalgesic Priming (neonatal incision, adult reincision) or (BDNF/IL-6, PGE2)	Mice	(108,159)

Estrogen	Spinal Cord	Greater allodynia and spinal pERK following model than females, transient hyperalgesia with intrathecal estrogen	OVX reduces allodynia and spinal pERK expression and prevents analgesic effect of ERK targeting drug in spinal cord	Hyperalgesic Priming (pH 4.0, pH 4.0)	Rats	(111)
Opioid Receptors	Spinal Cord	Endogenous MOR, KOR and M/DOR heteromer inhibition demonstrated with LS. DOR inhibiton found in neuropathic but not incision model	Endogenous MOR, KOR and M/DOR heterome inhibition demonstrated with LS. DOR inhibiton found in neuropathic but not incision model. KOR inhibition greater in females	Latent sensitization (hindpaw inflamation or CINP, opioid block)	Mice	(130,131)
Protein Kinase Mζ	Spinal Cord	Blocking or knockout prevents pain	Blocking or knockout doesn't prevent pain	Hyperalgesic priming (pH 4.0, pH 4.0)	Rats	(152)
Sestrin2	Spinal Cord	Increased expression after priming incision, exogenouse serstrin2 prevented hyperalgesia	Increased expression after priming incision, exogenouse serstrin2 prevented hyperalgesia	Hyperalgesic priming (neonatal incision, adult reincision)	Rats	(104)
Cap-dependent translation	Spinal Cord	Blockade prior to induction does not prevent hyperalgesia	Blockade prior to induction prevents hyperalgesia, estrogen dependent	Hyperalgesic priming (IL-6, PGE2)	Mice	(107)
Serotonin	Rostroal ventromedial Medulla	Gonadectomy does not impact SERT or 5HT2A expression. SERT inhibition decreases pain	Exogenous testosterone decreases SERT and increases 5HT2A expression. SERT inhibition decreases pain	Hyperalgesic priming (pH 5.0, fatiguing muscle contractions + pH 5.0)	Mice	(171)
Testosterone	Rostral ventromedial medulla	Decreases widespread pain through aromatization to estrogen and ERalpha receptors	Decreases widespread pain through SERT reduction in RVM	Hyperalgesic priming (pH 5.0, fatiguing muscle contractions + pH 5.0)	Mice	(25,170,171)
Atypical Protein Kinase C	Amygdala	Blockade prevents hyperalgesia, upregulated after priming	Blockade prevents hyperalgesia to a lesser	Hyperalgesic Priming (incision, PGE2)	Mice	(109)

			degree than in males, not upregulated after priming			
AMPA Receptors	Amygdala	GluA2 blockade reduces priming, upregulated following priming	GluA2 blockade reduces priming, upregulated following priming	Hyperalgesic Priming (incision, PGE2)	Mice	(109)
Mu Opioid Receptors	Amygdala	CeA non-specific opioid block or MOR block reinstates hyperalgesia	CeA non-specific opioid block does not reinstate hyperalgesia, but MOR block does	Latent sensitization (hindpaw inflamation, opioid block)	Mice	(165)
Somatostatin Expressing Neurons	Amygdala	Inhibition prevents priming for chronic pain, increased activity in pain states	Inhibition prevents priming for chronic pain, increased activity in pain states	Hyperalgesic priming (pH 4.0, pH 4.0)	Mice	(166)
T-type Calcium Channels	Thalamus	Activates ERK and facilitates development of pain. Brain directed blockade prevents development of pain	Activates ERK and facilitates development of pain. Brain directed blockade prevents development of pain	Hyperalgesic priming (pH 4.0, pH 4.0)	Mice	(167)
